# Electronic transparency of internal interfaces in metallic nanostructures comprising light, heavy and ferromagnetic metals measured by terahertz spectroscopy

**DOI:** 10.1515/nanoph-2023-0721

**Published:** 2024-01-15

**Authors:** Nicolas S. Beermann, Savio Fabretti, Hassan A. Hafez, Maria-Andromachi Syskaki, Iryna Kononenko, Gerhard Jakob, Mathias Kläui, Dmitry Turchinovich

**Affiliations:** Fakultät für Physik, Universität Bielefeld, Universitätsstr. 25, 33615 Bielefeld, Germany; Institute of Physics, Johannes Gutenberg Universität Mainz, Staudinger Weg 7, 55128 Mainz, Germany

**Keywords:** terahertz, conductivity, metallic nanostructures, spintronics, transport

## Abstract

We investigate the electronic transport at the internal interface within a selection of metallic bilayer nanostructures using the contact-free, all-optical method of THz time-domain spectroscopy. The Ru/Co, Ru/Pt, and Ru/Al bilayer nanostructures and their individual constituent metals are studied, with Ru representing an archetypal *d*-band metal, Co an archetypal ferromagnet, and Pt and Al archetypal heavy and light metals, respectively. The THz conductivity data were analyzed in terms of Drude and Bloch–Grüneisen models, and the interface current coefficient of the internal nanointerface was determined. Strong temperature dependency of the interface current coefficient in the Ru/Co nanostructure is revealed.

## 1 Introduction

Metallic nanostructures and thin films have become essential in modern science and technology, playing a fundamental role in various scientific and technological advances over the past decades [1], [2]. Such nanostructures offer unique capabilities, enabling light–matter interaction at the nanoscale [3] with applications in sensing [4], [5], imaging [6] and electronics [7], among others. Further, layered metallic nanosized systems exhibit enhanced mechanical [8] and magneto-resistive properties [9] that serve as fundamental components for modern spintronic devices [10], [11], [12].

For all applications mentioned above, and especially so for spintronics, the interface between two functional layers plays a fundamental role in governing the electronic properties of a nanodevice. This motivates experiments to obtain a deep understanding of electron transmission across the nanointerface. However, directly measuring the internal interface properties can be challenging [13] and relies on conventional transport measurement methods. These methods require electrical contacts and can introduce complications that may affect the measurement itself [14].

In a prior study [15], a method has been developed to quantify the electronic transparency between the two adjacent thin conducting layers using an all-optical and contact-free method of THz time-domain spectroscopy (THz-TDS). This methodology establishes a basis for a quantitative study of interfaces, creating opportunities for further research.

In this work, we expand on the previous analysis of internal interfaces by investigating three different metallic bilayers, Ru/Co, Ru/Pt, and Ru/Al, as well as their constituent thin film components. The choice of materials was motivated by the fact that Ru is an archetypal *d*-band metal, Co an archetypal ferromagnet, and Pt and Al archetypal heavy and light metals, respectively [16]–[20]. Particularly, Ru is an interesting material that is widely used in spintronics as a coupling layer and has recently been predicted to exhibit a large orbital Hall effect [21]. The comparison between these samples will highlight the impact that the metallic composition has on the electronic transparency of the internal interface within the nanostructure. Furthermore, we demonstrate the strong temperature-dependent behavior of the conductivity for the Ru/Co bilayer in the temperature range of 10–300 K. This reveals the thermal influence on the electronic transport for the whole metallic bilayer and emphasizes the role of the internal interface for the overall in-plane conduction mechanism.

## 2 Experiment

The examined metallic structures were fabricated by DC-magnetron sputtering on 500 µm thick MgO (100) substrates. The metallic structures in this study are Ru_*x*_/Co_*x*_, Ru_*x*_/Pt_*x*_, and Ru_*x*_/Al_*x*_, each individual layer having a thickness of *x* = 10 nm. For comparability, each one of the four constituent metals was also produced as single-element sample with 10 nm layer thickness. All our samples were capped by a 2 nm MgO layer. This serves as a protective coating for the metals to prevent oxidation and to ensure a uniform and symmetrical external interface of the thin films. The samples were additionally covered with a non-conductive 2 nm HfO_2_ cap layer to prevent degradation due to water absorption of the MgO layer. The bare MgO substrate, used as a reference in THz spectroscopy, was also covered with the 2 nm HfO_2_ cap layer. No measurable change in the THz dielectric properties of the substrate was observed due to the presence of this cap layer (see Supplementary Note 1).

A commercially available THz time-domain spectrometer (“TeraFlash Pro” from Toptica Photonics AG), providing a useful spectroscopy bandwidth of 0.3–2.5 THz, was used in transmission-mode in our THz-TDS measurements [22]. The electric field component of the THz radiation induces net in-plane electric currents inside the metallic film, which obey Ohm’s law, leading to frequency-dependent dissipation and phase delay of the transmitted THz signal. The measured THz transmission spectrum reveals the complex-valued and frequency-dependent conductivity of the entire sample, giving insights into the overall current distribution within these structures. All measurements were conducted at normal incidence with the THz beam focused onto the sample surface. The THz beampath within the spectrometer was purged with dry air to prevent the THz absorption by atmospheric water. For temperature-dependent measurements, the samples were mounted inside a closed-cycle helium cryostat installed at the THz focus position, enabling measurements at different sample temperatures in the range of *T* = 10 − 300 K. The cryostat was equipped with two THz-transparent quartz windows.

First, the refractive index *n*_s_ of a bare MgO substrate with a 2 nm HfO_2_ cap layer was characterized in the frequency range of *f* = 0.3 − 2.5 THz at different temperatures between 10 and 300 K. No measurable absorption of THz light was detected, and the refractive index exhibited a normal dispersion, well described by a second-order polynomial fitting function (see Supplementary Note 2). The precise knowledge of the substrate refractive index is needed for extraction of the sheet conductance spectra ${\tilde{\sigma }}_{\text{s}}\left(f\right)$ of the metallic thin films deposited on such a substrate using the Tinkham formalism [23], [24]. The data analysis procedure for calculating the conductance spectra and the applied corrections to account for possible substrate thickness variations are explained in detail in Supplementary Note 3.

## 3 Results and discussion

**Figure 1:**
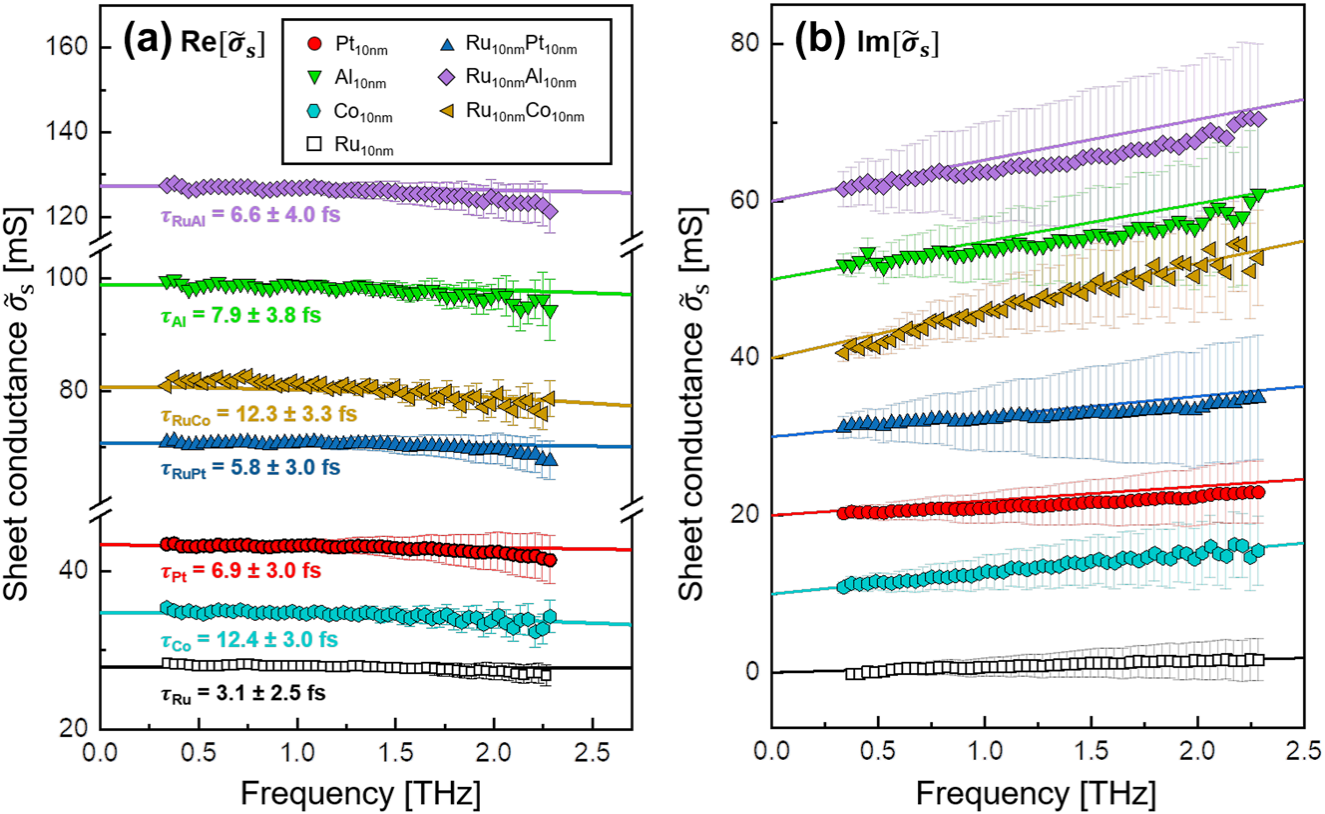
The complex sheet conductance spectra for the three metallic bilayers, Ru/Co, Ru/Pt, and Ru/Al, as well as their individual thin film components. Each layer has a thickness of 10 nm. The figure is divided into (a) real and (b) imaginary parts. For improved clarity, the imaginary parts are vertically shifted. The solid lines depict Drude fits and their corresponding electron scattering times are indicated in the graph.

Figure 1 shows the complex-valued sheet conductance spectra ${\tilde{\sigma }}_{\text{s}}\left(f\right)$ within the frequency range of *f* = 0.3 − 2.3 THz for all three examined metallic bilayers and their constituting thin films at room temperature. The 10 nm thick Ru thin film has the lowest sheet conductance, whereas the spectra of Ru/Al bilayer display the highest values.

The sheet conductance spectra were fitted with the classical free-carrier conduction Drude model [25], [26]

(1) $${\tilde{\sigma }}_{\text{s}}\left(\omega \right)=\tilde{\sigma }\left(\omega \right)d=\frac{\sigma_{\text{DC}}}{1-i\omega \tau }d,$$

where $\tilde{\sigma }\left(\omega \right)$ is the effective complex-valued conductivity of the whole structure, *σ*_DC_ denotes the DC-conductivity, *d* is the thickness of the conductive film, *ω* = 2*πf* is the angular frequency, and *τ* is the electron momentum scattering time. The best fitting for all thin metallic films, represented by the solid lines in Figure 1, is obtained with the fitting parameters summarized in Table 1. We have additionally indicated the thereby obtained scattering times *τ* in Figure 1(a).

**Table 1:** Drude fitting parameters *σ*_DC_ and *τ* for single-element thin films Ru, Co, Pt, and Al, as well as metallic bilayer structures Ru/Co, Ru/Pt, and Ru/Al at room temperature. Each respective layer has a thickness of 10 nm.

Sample	*σ*_DC_ [MS/m]	*τ* [fs]
Ru_10nm_	2.8 ± 0.2	3.1 ± 2.5
Co_10nm_	3.5 ± 0.2	12.4 ± 3.0
Pt_10nm_	4.3 ± 0.2	6.9 ± 3.0
Al_10nm_	9.9 ± 0.2	7.9 ± 3.8
Ru_10nm_Co_10nm_	4.0 ± 0.2	12.3 ± 3.3
Ru_10nm_Pt_10nm_	3.5 ± 0.2	5.8 ± 3.0
Ru_10nm_Al_10nm_	6.4 ± 0.2	6.6 ± 4.0

The scattering times vary in the range of *τ* = 3.1 − 12.4 fs across all investigated metallic thin films, and the DC-conductivity ranges as *σ*_DC_ = 2.8 − 9.9 MS/m. Interestingly, both the Co monolayer and Ru/Co bilayer samples show longer electron scattering times than all other metallic structures comprising Al or Pt.

With the established sheet conductance spectra in Figure 1, we analyze the internal interface contribution to the overall in-plane conductivity within a bilayer nanostructure. Under the hypothesis that the bilayer can be described by a model in which the overall sheet conductance of the structure is the algebraic sum of sheet conductances of its individual layers, the influence of the internal interface on the electronic transport for the entire structure should vanish. However, for nanosized layers, the internal interface can modify the in-plane conductivity of the whole structure, providing for an additional contribution to its conductivity, as described in Ref. [15].

**Figure 2:**
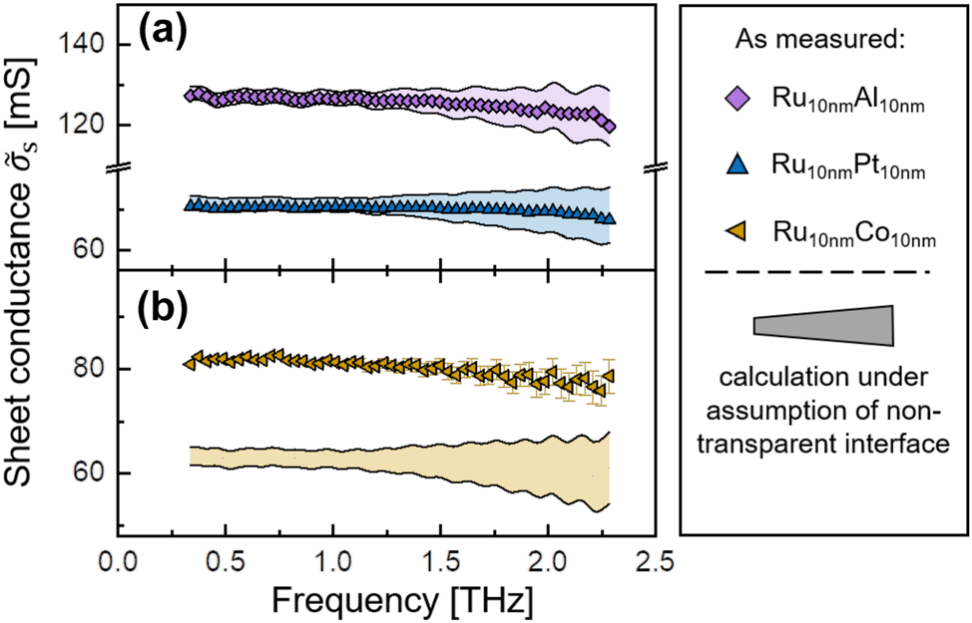
The real part of sheet conductance spectra for (a) Ru/Pt and Ru/Al and (b) Ru/Co as measured at room temperature (symbols). The colored area shows the calculation under the assumption of a non-transparent internal interface. For Ru/Pt and Ru/Al that assumption agrees with the obtained sheet conductance spectra, but the measured conductance of the Ru/Co bilayer surpasses the calculation.

In Figure 2, we show the comparison between the measured real part of the bilayer conductance (symbols) and the calculations under the assumption of a non-transparent internal interface (colored area). This calculation is based on the summation of the measured sheet conductances of individual thin film components. Figure 2(a) shows that the overall conductances of the Ru/Pt and Ru/Al metallic bilayers are well described by the summation of their individual metal thin film sheet conductances. This implies that the assumption of a non-transparent internal interface is applicable for these two bilayers. However, the conductance of Ru/Co bilayer does not agree with this assumption and its conductance exceeds the prediction. This result for the Ru/Co bilayer is consistent with the earlier study in Ref. [15] and corresponds to the situation of a partially-transparent internal interface within the bilayer, as briefly explained below.

**Figure 3:**
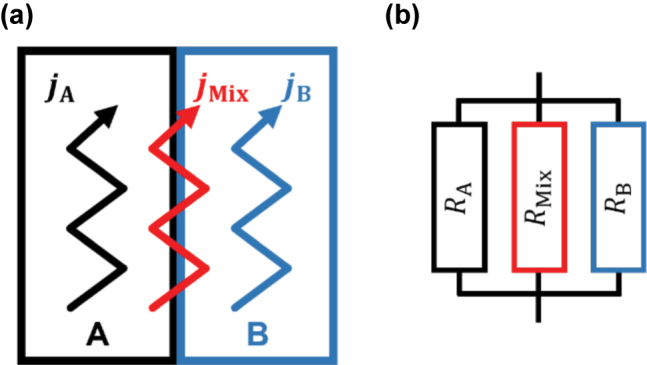
Illustration of the internal interface model for the metallic bilayers. Subfigure (a) shows the three THz-driven currents inside the metallic bilayer, consisting of the currents *j*_A_, *j*_B_, and *j*_Mix_; and subfigure (b) depicts an effective resistor network corresponding to the current pathways.

The bilayer system is schematically illustrated in Figure 3(a) and comprises three distinct THz-driven electronic currents. The first two currents, *j*_A_ and *j*_B_, flow independently in their respective layer section of the bilayer and reflect their individual conductive properties. Here, A and B stand for two different metal thin films that constitute the bilayer. The third current, here termed mixing current *j*_Mix_, emerges within the metallic bilayer due to the presence of an internal interface. It represents the current that cannot be assigned to one single respective layer within a bilayer structure and thus “sees” the entire structure. These current pathways correspond to the effective resistor network depicted in Figure 3(b), and the discrepancy between the conductances of the bilayer and its individual components is now expressed by

(2) $$\Delta {\tilde{\sigma }}_{\text{s},\text{AB}}\left(f\right)={\tilde{\sigma }}_{\text{s},\text{AB}}\left(f\right)-\left[{\tilde{\sigma }}_{\text{s},\text{A}}\left(f\right)+{\tilde{\sigma }}_{\text{s},\text{B}}\left(f\right)\right].$$

This conductance indicates the presence of the additional mixing current channel proportional to $\Delta {\tilde{\sigma }}_{\text{s},\text{AB}}\left(f\right)$ and, therefore describes the isolated conductance contribution of the interface to the transport through an entire bilayer structure. This enables the introduction of the interface current coefficient, ${\tilde{t}}_{\text{I}}\left(f\right)$ , which is calculated by comparing the mixing current in relation to the independently flowing currents in the two separate layers [15]

(3) $${\tilde{t}}_{\text{I}}\left(f\right)=\frac{j_{\text{Mix}}}{j_{\text{A}}+j_{\text{B}}}=\frac{\Delta {\tilde{\sigma }}_{\text{s},\text{AB}}\left(f\right)}{{\tilde{\sigma }}_{\text{s},\text{A}}\left(f\right)+{\tilde{\sigma }}_{\text{s},\text{B}}\left(f\right)}.$$

This complex-valued, frequency-dependent coefficient describes the significance of the internal interface for the overall in-plane conduction behavior of the metallic bilayer. The interface current coefficient provides insight into the electronic transparency of the internal interface, with a higher coefficient suggesting an increased electronic transparency. When the mixing current vanishes for the entire structure, the interface sheet conductance $\Delta {\tilde{\sigma }}_{\text{s},\text{AB}}\left(f\right)$ and the interface current coefficient also vanish. In this situation, the effective resistor circuit now comprises only two parallel resistors, which is electrically equivalent to a situation where the two layers are either composed of the same material or are spatially separated.

In contrast to the Ru/Co bilayer, the Ru/Al and Ru/Pt samples exhibited no additional contribution from the internal interface, hence a vanishing ${\tilde{t}}_{\text{I}}\left(f\right)$ (see Figure 6(c)). Now we will study the effect of temperature on the degree of electronic transparency of internal interface in the Ru/Co bilayer, for which we also need to establish the temperature-dependent conductivities of its individual constituents Ru and Co.

**Figure 4:**
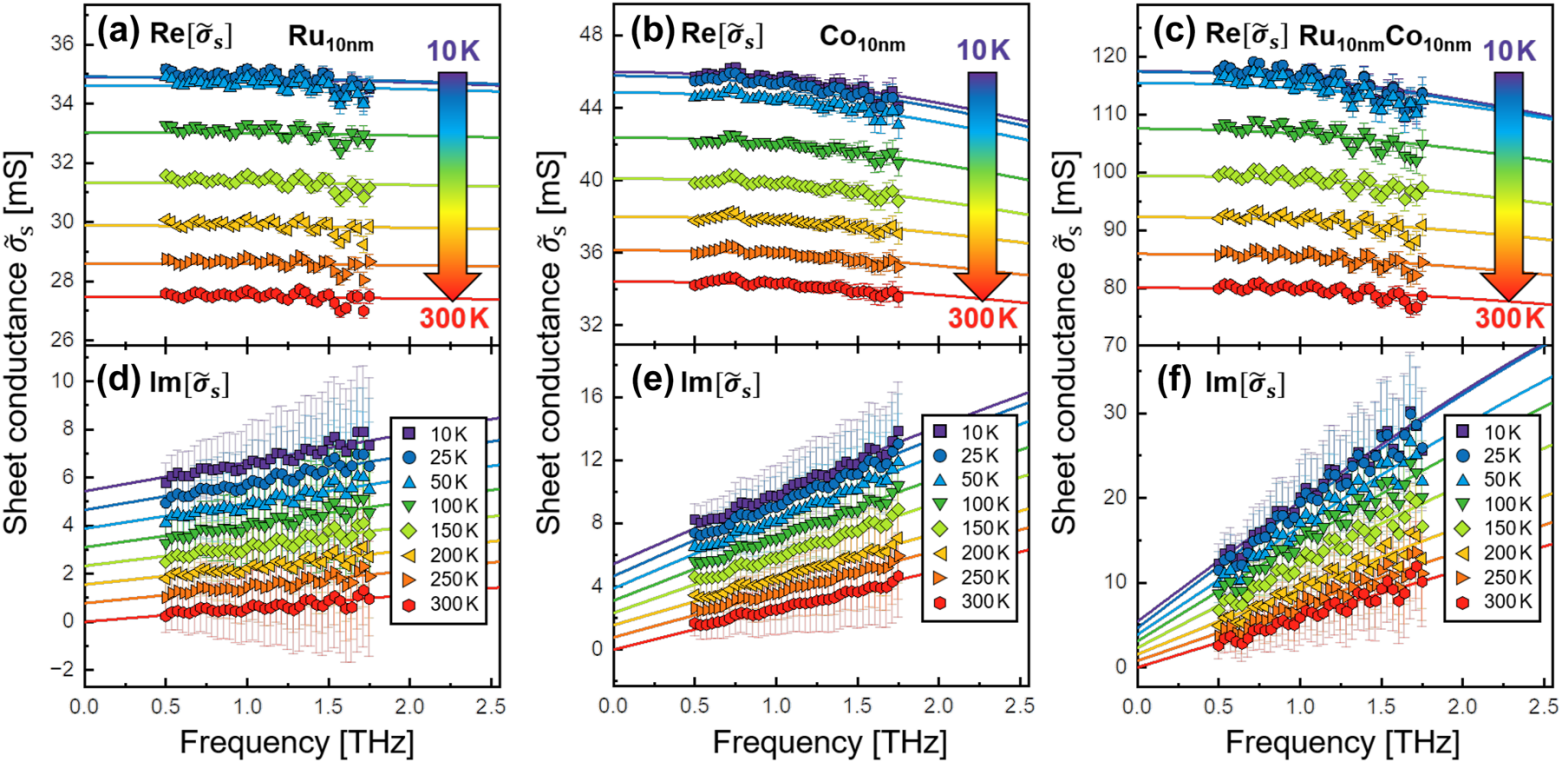
The complex sheet conductance spectra for the Ru, Co, and Ru/Co thin films at temperatures ranging from 10 to 300 K. The spectra are divided into real (a–c) and imaginary parts (d–f). The large arrows highlight the temperature increase, which correlates with a decrease in conductivity. For improved clarity, all imaginary parts are vertically shifted. The solid lines represent Drude fits.

In Figure 4, the sheet conductance spectra ${\tilde{\sigma }}_{\text{s}}\left(f\right)$ for the Ru, Co, and Ru/Co metallic thin films in the temperature range of 10–300 K are shown. They are subdivided into real (a–c) and imaginary parts (d–f). For all three metallic samples, similar behavior is observed. The decrease of temperature leads to an increase of conductivity, which saturates at temperatures below 50 K. This temperature dependency of the conductivity is commonly observed in metals [27]. Figure 4 also shows that the sheet conductance spectra are still well described by the Drude model. These spectra will assist us in determination of the temperature dependency of the electronic transparency of the internal Ru/Co interface. The isolated interface conductance for the Ru/Co bilayer, $\Delta {\tilde{\sigma }}_{\text{s},\text{RuCo}}\left(f\right)$ , and its corresponding Drude fit are presented in Supplementary Note 4 (see also Ref. [15]).

**Figure 5:**
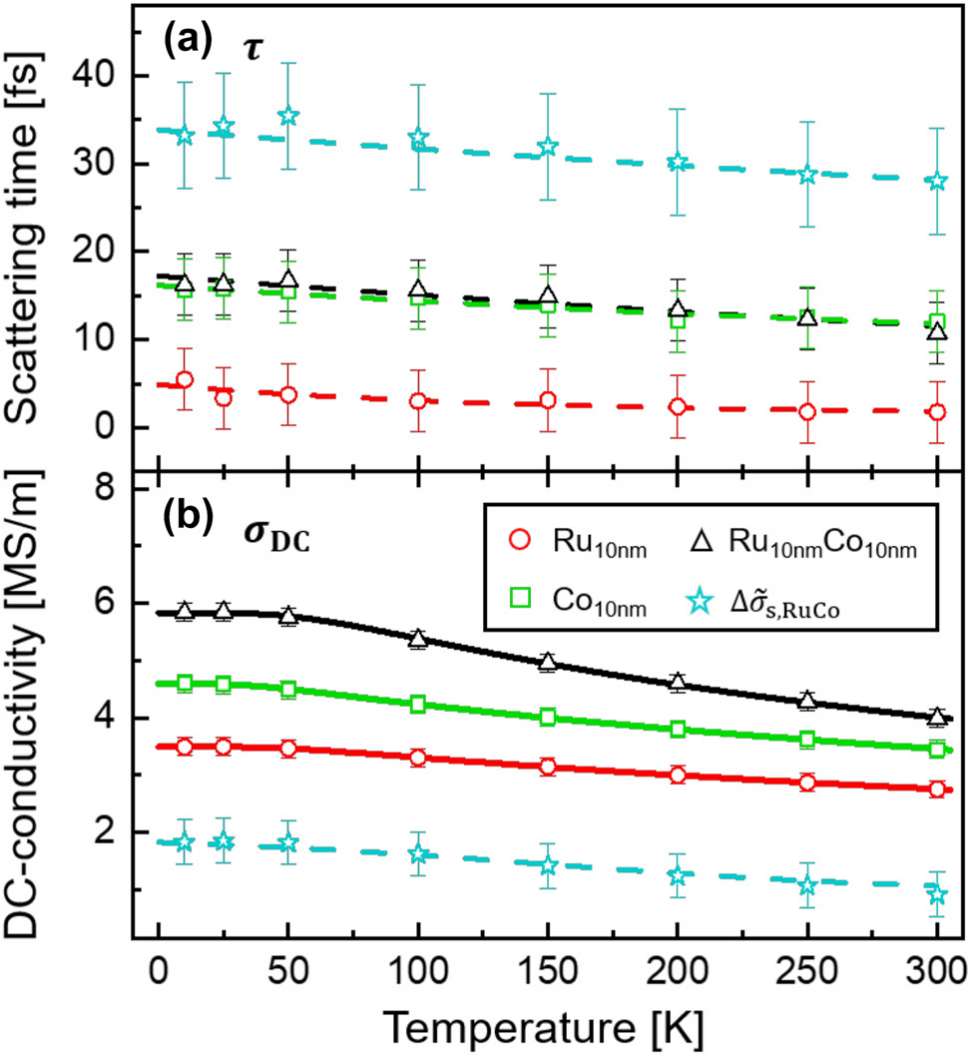
The temperature-dependent Drude fitting parameters for the Ru, Co, and Ru/Co thin films, as well as for $\Delta {\tilde{\sigma }}_{\text{s},\text{RuCo}}$ , for (a) electron momentum scattering time *τ* and (b) the DC-conductivity *σ*_DC_, respectively. The dashed lines are guides to the eye, whereas the solid lines are conductivity fits using the Bloch–Grüneisen (BG) formalism.

The obtained temperature-dependent Drude fitting parameters for the three samples are depicted in Figure 5. Similar to the temperature dependency for the sheet conductance spectra discussed above, both the electron momentum scattering time (shown in Figure 5(a)) and the DC-conductivity (shown in Figure 5(b)) increase as the sample is cooled down and then saturate for temperatures below 50 K. This can be attributed to a reduced phonon population caused by the lower thermal energy and consequently an increase of the electron mean free path for lower temperatures [28], [29]. The Drude fitting parameters for the interface conductance spectra $\Delta {\tilde{\sigma }}_{\text{s},\text{RuCo}}\left(f\right)$ are also shown in Figure 5. This additional contribution exhibits a longer scattering time, but lower DC-conductivity, as compared to those of the Ru and Co monolayers and the Ru/Co bilayer.

Under the assumption of a constant speed of sound, the temperature dependency of the conductivity for metals can be analytically described by the Bloch–Grüneisen (BG) theory [30], [31]

(4) $$\sigma \left(T\right)={\left[\rho_{0}+A{\left(\frac{T}{\Theta_{\text{D}}}\right)}^{5}\int_{0}^{\Theta_{\text{D}}\;/T}\frac{x^{5}}{\left(e^{x}-1\right)\left(1-e^{-x}\right)}dx\right]}^{-1},$$

where *ρ*_0_ corresponds to the temperature-independent residual resistivity, *A* is a material-specific constant, and Θ_D_ is the Debye temperature of the metal. This formalism has been successfully utilized for the electrical characterization of nanoscale metallic films [32], [33], [34], [35]. Here, we apply this formalism to our DC-conductivity data. The results are illustrated in Figure 5(b) by the solid lines, and the corresponding fitting parameters are listed in Table 2.

**Table 2:** BG fitting parameters *ρ*_0_, *A*, and Θ_D_ for the Ru, Co, and Ru/Co metallic thin films. The Debye temperature bulk literature values for Ru and Co are also presented.

Sample	*ρ*_0_ [μΩ cm]	*A* [μΩ cm]	Θ_D_ [K]	Lit. Θ_D_ [K]
Ru_10nm_	28.7 ± 0.5	35.5 ± 22.5	324 ± 182	555 [39]
Co_10nm_	21.8 ± 0.6	23.6 ± 21.0	238 ± 203	460 [39]
Ru_10nm_Co_10nm_	17.2 ± 0.5	42.5 ± 23.9	373 ± 178	–

The established Debye temperatures for the Ru and Co thin films are reduced by ≈42% and ≈48% for Ru and Co, respectively, as compared to the bulk literature values, which are also shown in Table 2. Such a reduction in the Debye temperature for thin films is consistent with findings reported for other metallic nanostructures, including gold thin films [32], [33] or copper wires [36]. This decrease of the Debye temperature for nanosized structures has been attributed to a reduced coordination number of atoms at the surface, defects, and grain boundaries, which causes surface phonon softening [29], [33].

**Figure 6:**
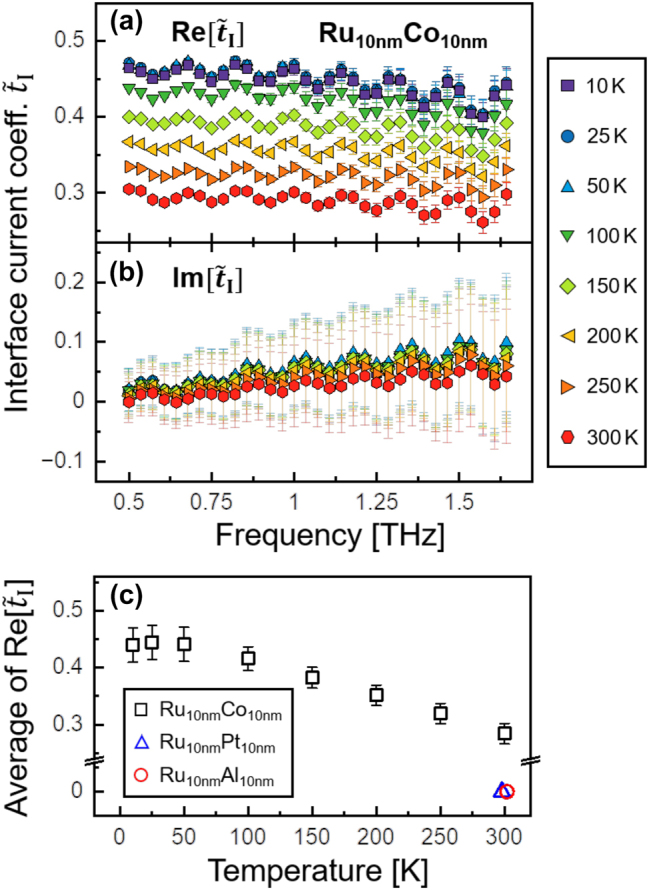
The complex-valued interface current coefficient ${\tilde{t}}_{\text{I}}\left(f\right)$ of the internal Ru/Co interface is illustrated in the temperature range of *T* = 10 − 300 K, split into (a) real and (b) imaginary parts. Subfigure (c) highlights the frequency-averaged real part of the coefficient for all three investigated bilayers.

Finally, the internal interface current coefficient ${\tilde{t}}_{\text{I}}$ is calculated from the measured temperature-dependent Ru/Co sheet conductance spectra and is presented in Figure 6. This coefficient is predominantly real-valued and exhibits minimal dispersion within the range of *f* = 0.5 − 1.8 THz. The real part $\text{Re}[{\tilde{t}}_{\text{I}}$ ] is presented in Figure 6(a) and shows a value of $\text{Re}\left[{\tilde{t}}_{\text{I}}\right]\approx 0.28\pm 0.02$ at room temperature, which matches the value reported before [15]. This value increases monotonically as the temperature decreases until it reaches a constant value of $\text{Re}\left[{\tilde{t}}_{\text{I}}\right]\approx 0.44\pm 0.03$ for temperatures below *T* = 50 K. This represents a significant 57 % increase compared to the value at *T* = 300 K. The imaginary parts $\text{Im}\left[{\tilde{t}}_{\text{I}}\right]$ shown in Figure 6(b) are very small and show no clear temperature dependency. Figure 6(c) depicts the frequency-averaged real part of the interface current coefficient for all three investigated bilayer structures. The interface coefficient for the Ru/Co bilayer is significant and shows a linear increase as the structure is cooled down. Below 50 K, the values of this coefficient saturate.

The relatively high interface current coefficient in Ru/Co, as well as its temperature behavior, can be explained by the following. First, the transition of electrons between Co and Ru is favored by their similar crystal structure (both hcp). Their Fermi surfaces share the same symmetry [37], therefore enabling the electron transfer from one metal to another with conserved electron momentum. Also, during growth of the polycrystalline films, the surface of the Ru layer acts as a substrate for the isostructural Co layer, which minimizes grain boundary resistance contributions over the interface. Further, the mean free path of electrons in Ru and Co should be also taken into account. At room temperature, theoretical calculations provide characteristic mean free paths for bulk Co and Ru of approximately 12 nm and 7 nm [38], respectively. These are of the same order of magnitude as the thickness of the nanostructure itself. With decreasing temperatures of the sample, the mean free path for electrons within the structure increases due to the reduced phonon population [28], [29], leading to an increase in both scattering time and conductivity (as seen in Figure 5). Consequently, the mean free path becomes larger in relation to the dimensions of the structure, resulting in a transition in the conduction mechanism toward a more ballistic regime. As a result, the electrons experience less obstruction during their motion and can effectively transverse the internal interface, resulting in an increased interface current coefficient. Finally, the behavior of the interface coefficient at temperatures below 50 K mirrors the behavior of the measured conductivity, exhibiting saturation as the metal structure approaches its residual conductive state for low temperatures.

In contrast to a relatively transparent Ru/Co internal interface, the internal interface current coefficient for Ru/Pt and Ru/Al bilayers was found to be $\text{Re}\left[{\tilde{t}}_{\text{I}}\right]=0$ , measured at room temperature. This can be explained by a significant mismatch of the Fermi surfaces between the two constituent metals within the bilayer [37]: while both Al and Pt have fcc crystal structure, Ru has hcp structure. Correspondingly, the Fermi surfaces of Al and Pt have cubic symmetry while that of Ru has hexagonal symmetry, leading to a significant mismatch of the Fermi wave vectors at the interface.

## 4 Summary

In summary, this study demonstrates an analysis of the in-plane THz conductivity of technologically important Ru/Co, Ru/Pt, and Ru/Al bilayer nanostructures and their individual thin film components. The conductivity spectra of all samples could be effectively described by the classical Drude model, revealing the electron scattering times of the nanosized metal layers. The comparison between the THz spectra of bilayers and constituting thin films allowed us to quantitatively determine the interface current coefficient of internal metal–metal interface within the metallic bilayer nanostructures. While internal interfaces of Ru/Pt and Ru/Al bilayers were found to be electronically opaque, the interface current coefficient of the Ru/Co bilayer was significant and also strongly temperature dependent. Furthermore, the Debye temperature for Ru, Co, and Ru/Co nanostructures was estimated using the Bloch–Grüneisen formalism, showing a reduction of the Debye temperatures for nanofilms as compared to the bulk materials.

## Supplementary Material

Supplementary Material Details
