# Supplementary material for: Electronic transparency of internal interfaces in metallic nanostructures comprising light, heavy and ferromagnetic metals measured by terahertz spectroscopy
Source: Nanophotonics. 2024 Jan 15;13(10):1883–90. doi: 10.1515/nanoph-2023-0721 (PMC11501111; doi:10.1515/nanoph-2023-0721)
Supplement: Supplementary file 1 — Supplementary Material Details [file j_nanoph-2023-0721_suppl_001.pdf]

Supplementary Information

Electronic Transparency of Internal Interfaces in Metallic Nanostructures Comprising Light, Heavy and Ferromagnetic Metals Measured by Terahertz Spectroscopy

Nicolas S. Beermann[1], Savio Fabretti[1], Hassan A. Hafez[1], Maria-Andromachi Syskaki[2,3], Iryna Kononenko[2], Gerhard Jakob[2], Mathias Kläui[2] and Dmitry Turchinovich[1]

[1]*Fakultät für Physik, Universität Bielefeld, Universitätsstr. 25, 33615 Bielefeld, Germany*

[2]*Institut für Physik, Johannes Gutenberg Universität Mainz, Staudingwerweg 7, 55128 Mainz, Germany*

[3]*Singulus Technologies AG, 63796 Kahl am Main, Germany*

Supplementary Note 1: Refractive index comparison of uncoated and coated MgO (100) substrates

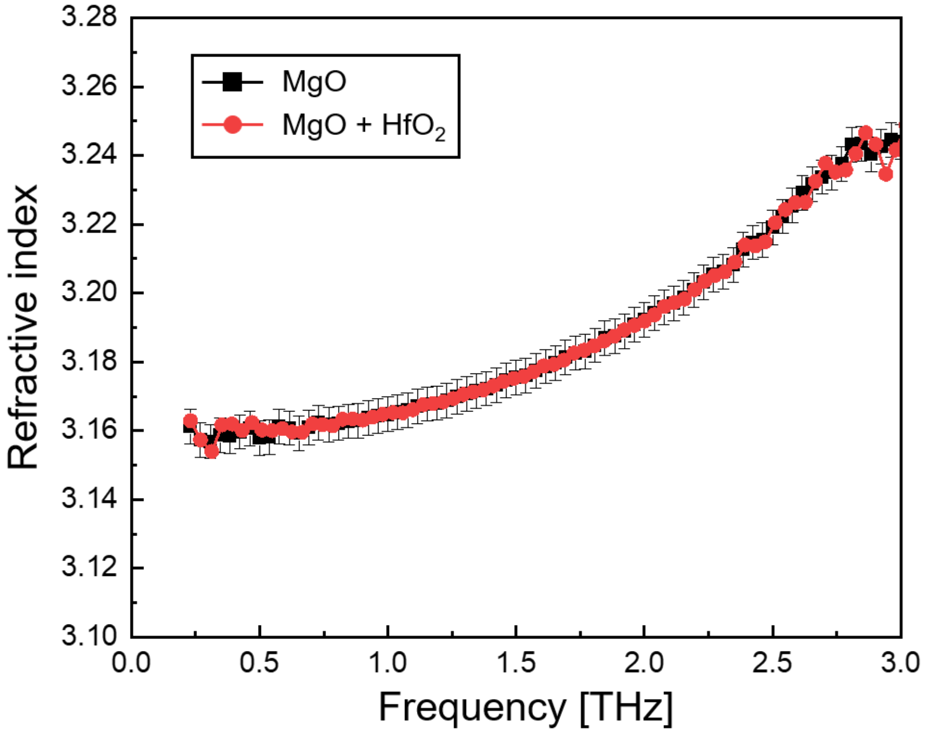

Fig. S1: *Illustrates the refractive index $n_s(f)$ comparison of a bare MgO substrate and the substrate coated with 2 nm HfO_2 cap layer. There is no significant change observable.*

We can determine the refractive index $n_s(f)$ of a non-conductive substrate using terahertz time-domain spectroscopy (THz-TDS). Fig. S1 shows the refractive index results of a bare 500 µm thick MgO (100) substrate (black squares) and another MgO substrate with an additional 2 nm HfO_2 cap layer on top (red circles). Within the frequency range of $f = 0.25 - 3$ THz, no change was observable. Consequently, the additional HfO_2 top layer has no influence on the refractive index of the substrate and can be neglected in the analysis.

Supplementary Note 2: Temperature-dependent refractive index of the 500 µm thick MgO (100) substrate

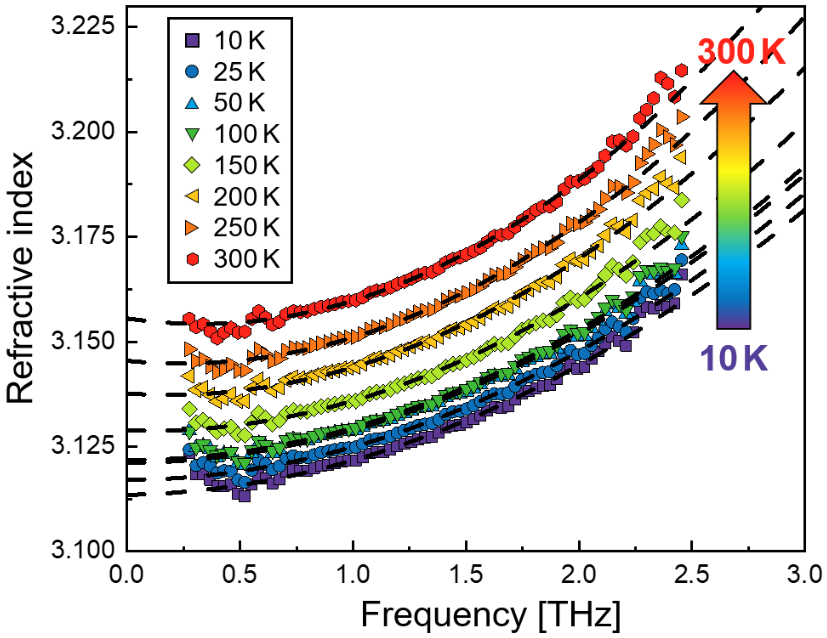

Fig. S2: *Refractive index of a MgO (100) substrate as a function of frequency for various temperatures in the range of* $T = 10 - 300$ K. *The dashed lines represent second-order polynomial fits.*

Fig. S2 displays the frequency-dependent refractive index $n_s(f)$ of a MgO (100) substrate for different temperatures. The refractive index tends to decrease for lower temperatures. To account for the dispersion of the substrate, the refractive index was fitted by a second-order polynomial function

$$n_s(f) = a + b \cdot f + c \cdot f^2, \quad \text{(S1)}$$

where f is the frequency in THz units. This fit was established for every temperature in the frequency range of $f = 0.25 - 2.5$ THz. The resulting parameters are summarized in Tab. S1.

Tab. S1: *Summary for the polynomial dispersion fits of MgO.*

T **[K]**	***a***	***b*** **[THz^{-1}]**	***c*** **[THz^{-2}]**
10	3.114	$2.878 \cdot 10^{-4}$	$7.518 \cdot 10^{-3}$
25	3.117	$-2.445 \cdot 10^{-4}$	$7.729 \cdot 10^{-3}$
50	3.121	$-1.241 \cdot 10^{-4}$	$7.716 \cdot 10^{-3}$
100	3.122	$-7.978 \cdot 10^{-4}$	$8.098 \cdot 10^{-3}$
150	3.129	$-2.101 \cdot 10^{-3}$	$8.899 \cdot 10^{-3}$
200	3.138	$-3.700 \cdot 10^{-3}$	$9.877 \cdot 10^{-3}$
250	3.145	$-5.149 \cdot 10^{-3}$	$1.082 \cdot 10^{-2}$
300	3.155	$-7.670 \cdot 10^{-3}$	$1.213 \cdot 10^{-2}$

Supplementary Note 3: Conductivity calculation via Tinkham formalism and substrate thickness correction

The metallic structures represent a thin THz-absorptive layer deposited on a non-conductive thick substrate. The absorptive film is orders of magnitude thinner than the incident THz light wavelength, causing many Fabry-Pérot reflections within the nanosized film that interfere constructively. Under this condition, this interference leads to a uniform THz electric field distribution across the thin film. Based on this Tinkham formalism, the complex THz transmission $\tilde{T}(f)$, which is obtained through the reference and sample measurement, can be related to the sheet conductance $\tilde{\sigma}_\mathrm{s}(f)$ of the thin film using the Tinkham equation [1], [2]

$$\tilde{T}(f) = \frac{\tilde{E}_\mathrm{sam}(f)}{\tilde{E}_\mathrm{ref}(f)} = \frac{1+n_\mathrm{s}(f)}{1+n_\mathrm{s}(f)+Z_0\,\tilde{\sigma}_\mathrm{s}(f)}. \quad \text{(S2)}$$

Here $\tilde{E}_\mathrm{sam}(f)$ and $\tilde{E}_\mathrm{ref}(f)$ are the frequency-dependent THz electric fields transmitted through the substrate with the metallic thin film (sample), and through the bare substrate (reference). Furthermore, $Z_0 = 377\,\Omega$ is the impedance of free-space and $n_\mathrm{s}(f)$ denotes the before mentioned refractive index of the bare MgO substrate. In the THz literature, the here introduced sheet conductance is commonly referred to as sheet conductivity. The thickness of the thin conductive film d links the complex-valued conductivity $\tilde{\sigma}(f)$ to the sheet conductance via $\tilde{\sigma}_\mathrm{s}(f) = \tilde{\sigma}(f)\,d$.

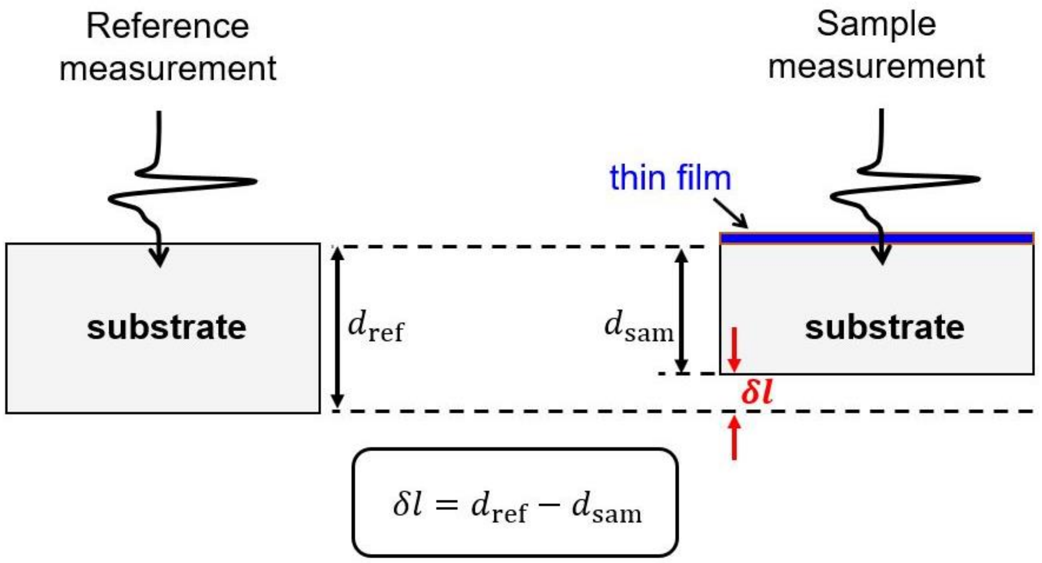

Fig. S3: *Schematic picture of the same substrate material but with different thicknesses for the reference and sample measurement. The small thickness difference is denoted as $\delta l = d_\mathrm{ref} - d_\mathrm{sam}$. This thickness variation is typically in the order of a few micrometers.*

Typically, two substrates of the same material are needed for the reference and sample measurement. In this case, one has to account for a possible minor thickness difference $\delta l = d_\mathrm{sam} - d_\mathrm{ref}$ between them, d_sam and d_ref are the respective substrate thicknesses. This is illustrated in Fig. S3. This thickness mismatch results to non-identical propagation times for the THz emission through each substrate. This temporal difference can be observed directly in the measured THz electric fields, as demonstrated in Fig. S4. The time between the peak of the main pulse and the corresponding etalon for the bare substrate is named Δt_ref, analogous this

time is called Δt_{sam} for the sample measurement. These times are established by interpolating the peak-position. The thickness of each substrate can now be estimated with

$$d = \frac{c_0}{2\, n_{\mathrm{s}}} \Delta t, \qquad \text{(S3)}$$

where c_0 represents the speed of light in vacuum and n_{s} the refractive index of the substrate. This allows to calculate the thickness difference between the substrates $\delta l = d_{\mathrm{sam}} - d_{\mathrm{ref}}$.

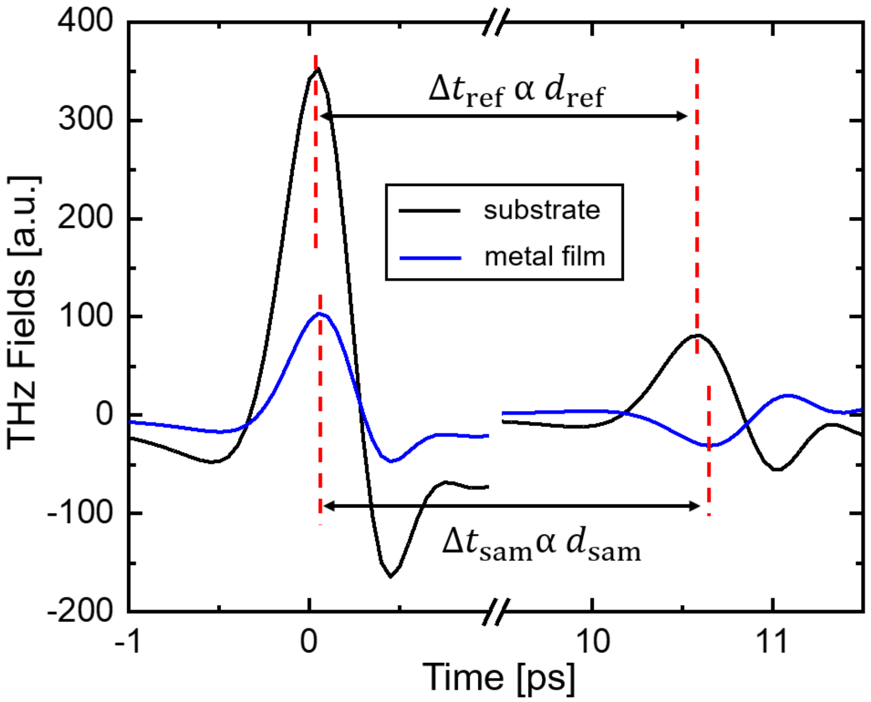

Fig. S4: *As an example, this figure shows the electric fields for the substrate (black line) and metal film (blue line) measurements. The thickness difference between the substrates leads to a temporal shift in the etalon signals. Therefore, Δt_{sam} and Δt_{ref} will be non-identical.*

The thickness difference δl is considered in the Tinkham equation for the THz transmission spectrum $\tilde{T}(f)$ with a frequency-dependent phase correction term

$$\tilde{T}(f) = \frac{\tilde{E}_{\mathrm{sam}}(f)}{\tilde{E}_{\mathrm{ref}}(f)} = \frac{1+n_{\mathrm{s}}(f)}{1+n_{\mathrm{s}}(f)+Z_0\, \tilde{\sigma}_{\mathrm{s}}(f)} e^{-i\,\varphi(f)}\,, \qquad \text{(S4)}$$

where $\varphi(f) = [n_{\mathrm{s}}(f) - 1]\, \delta l\, \frac{2\,\pi\, f}{c_0}$ is the phase originating from the thickness difference. In the case that the two substrates have the exact same thickness ($\delta l = 0$), then the exponential function vanishes. Solving equation (S4) for the sheet conductance $\tilde{\sigma}_{\mathrm{s}}(f)$ equates to

$$\tilde{\sigma}_{\mathrm{s}}(f) = \frac{1+n_{\mathrm{s}}(f)}{Z_0} \left[\frac{e^{-i\,\varphi(f)}}{\tilde{T}(f)} - 1\right]. \qquad \text{(S5)}$$

The determination of the substrate thickness and the respective thickness difference δl is constrained by both the temporal resolution of electric field measurements and the precision of interpolating the peak field positions. The uncertainty for δl introduces a systematic error that needs to be considered for the sheet conductance spectra. This error is proportional to the derivative of eq. (S5) with respect to the variable δl, resulting in the expression:

$$\Delta\tilde{\sigma}_{\mathrm{s}} \propto \frac{\partial \tilde{\sigma}_{\mathrm{s}}}{\partial(\delta l)} = \frac{1+n_{\mathrm{s}}(f)}{Z_0} \left(\frac{-i\,(n_{\mathrm{s}}(f)-1)\, 2\,\pi\, f\, e^{-i\,\varphi(f)}}{c_0\, \tilde{T}(f)}\right). \qquad \text{(S6)}$$

Consequently, the error bar of part of the sheet conductance spectra will increase with frequency.

Supplementary Note 4: Isolated conductance contribution of the internal interface $\Delta\tilde{\sigma}_{\mathrm{s,RuCo}}(f)$ for the Ru/Co bilayer

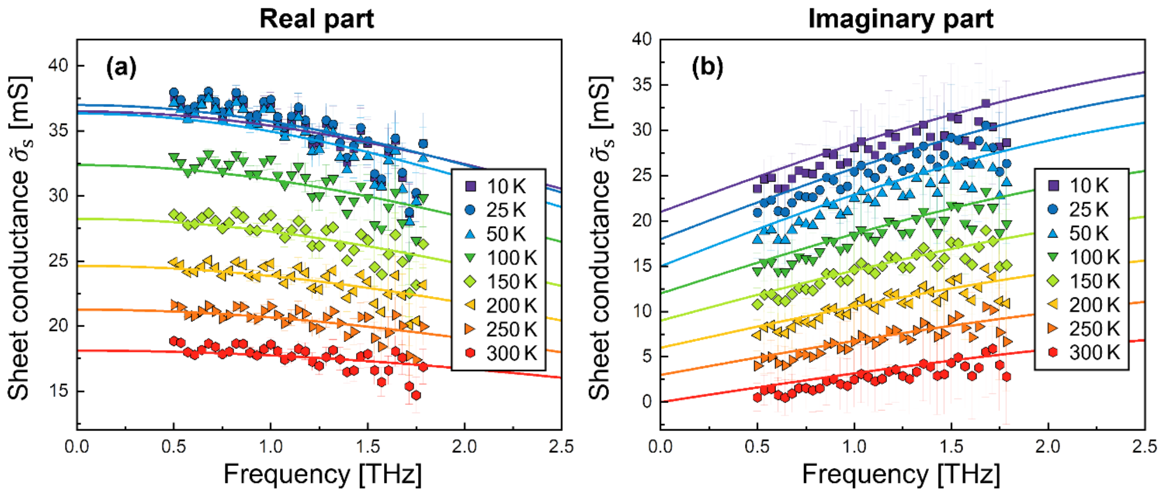

Fig. S5: *Illustrates the sheet conductance contribution of the internal interface for the Ru/Co bilayer, separated into* ***(a)*** *real and* ***(b)*** *imaginary part, respectively. The solid lines represent Drude fits and the imaginary parts are vertically shifted for improved clarity.*

Once the sheet conductance spectra for the Ru, Co and Ru/Co thin films are established, a comparison can be made between the bilayer and the sum of its individual components. This comparison involves calculating $\Delta\tilde{\sigma}_{\mathrm{s,RuCo}}(f)$ by subtracting the combined sheet conductances of Ru and Co from the sheet conductance of the Ru/Co bilayer

$$\Delta\tilde{\sigma}_{\mathrm{s,RuCo}}(f) = \tilde{\sigma}_{\mathrm{s,RuCo}}(f) - [\tilde{\sigma}_{\mathrm{s,Ru}}(f) + \tilde{\sigma}_{\mathrm{s,Co}}(f)]. \quad \text{(S5)}$$

In Fig. S5, the result for $\Delta\tilde{\sigma}_{\mathrm{s,RuCo}}(f)$ is illustrated for different temperatures between $T = 10 - 300$ K. The conductivity tends to increase for lower temperatures. These spectra are also fitted with the Drude model and their fit parameters are presented in Fig. 5 of the main text.

References

[1] R. E. Glover and M. Tinkham, “Conductivity of superconducting films for photon energies between 0.3 and 40kTc,” *Physical Review*, vol. 108, no. 2, pp. 243–256, 1957, doi: 10.1103/PhysRev.108.243.

[2] M. Tinkham, “Energy Gap Interpretation of Experiments on Infrared Transmission through Superconducting Films,” *Physical Review*, vol. 104, no. 3, pp. 845–846, Nov. 1956, doi: 10.1103/PhysRev.104.845.
